# Validity and reliability of a food frequency questionnaire for community dwelling older adults in a Mediterranean country: Lebanon

**DOI:** 10.1186/s12937-022-00788-8

**Published:** 2022-06-18

**Authors:** Nathalie Yaghi, Christa Boulos, Rafic Baddoura, Marianne Abifadel, Cesar Yaghi

**Affiliations:** 1 Department of Nutrition & Dietetics, Faculty of Pharmacy, Saint Joseph University of Beirut, PO Box: 17-5208 Mar Mikhael, Beirut, 1104 2020 Lebanon; 2 Faculty of Medicine, Saint Joseph University of Beirut, Beirut, Lebanon; 3 Laboratory of Biochemistry and Molecular Therapeutics, Faculty of Pharmacy, Pôle Technologie-Santé, Saint Joseph University of Beirut, Beirut, Lebanon; 4 Department of Gastroenterology, Faculty of Medicine, Saint Joseph University of Beirut, Beirut, Lebanon; 5 Hotel-Dieu de France University Hospital of Beirut, Beirut, Lebanon

**Keywords:** Validity, Reproducibility, Food frequency questionnaire, Dietary recall, Mediterranean population, Elderly

## Abstract

**Background:**

Food frequency questionnaires (FFQ) is an easy and inexpensive tool that can be used to evaluate nutrient and dietary trends of groups and individuals. Few studies in the East Mediterranean region tailored FFQs to describe dietary intakes of older adults. The purpose of the study is therefore to assess the validity and reproducibility of a FFQ, designed for use with older adults living in a Mediterranean Arabic speaking country, Lebanon.

**Methods:**

The FFQ is composed of a list of 90 food items, commonly consumed by adults above 60 years of age. Validity of the FFQ was tested using the mean of two 24-hours dietary recalls (24HDR), and reproducibility, by repeating the questionnaire within a one-month period, along the second dietary recall. Our study included 42 and 76 participants, for the repoducibility and validity analysis respectively. Subjects were randomly selected from 2 of the 8 governorates in the country.

**Results:**

FFQ reproducibility showed a mean relative difference of 1.03% without any significant difference between all paired components of nutrients. Intra class correlation (ICC) showed good and excellent reliability for caloric intake and all macronutrients, moderate to good reliability for all remaining nutrients, except for poly-unsaturated fatty acids, vitamins A, B12 and fibers. Correlation coefficients for all nutrients were fair to strong. Both administrations of the FFQ showed good internal validity. Validation of FFQ showed a mean relative difference between FFQ and mean 24HDR at 19.5%. Agreements between the 2 methods, for classifying individuals in the same or adjacent quartile, for nutrient intake and nutrient adequacy, were 80 and 78.2% respectively. Mean Kappa coefficient was 0.56 and energy-adjusted correlations were within the recommended values for all items except for vitamin A and B12. Adjusting for nutrient-dense food intake improved the agreement for theses 2 vitamins to 0.49 and 0.56, respectively.

**Conclusion:**

The proposed FFQ can be considered a valid tool to help describe nutrient intake of older individuals in an Arabic speaking Mediterranean country. It could serve for possible use in the East Mediterranean region for the evaluation of regular dietary intake of community-dwelling older adults.

**Supplementary Information:**

The online version contains supplementary material available at 10.1186/s12937-022-00788-8.

## Background

Understanding the impact of dietary factors on the development of chronic diseases in elderly, is important for better prevention and treatment [1–6]. Choosing the appropriate tools to evaluate nutrient intakes is therefore crucial and needs to be adapted to this age-group. In the general population, multiple tools are used to describe and reflect dietary habits and nutritional status of individuals.

Frequently used direct retrospective methods are diet records (DR), 24 hours dietary recalls (24HDR) and food frequency questionnaires (FFQ). They can be self-reported or interviewer- reported depending on the needs of the study. To reflect chronic and habitual intakes, it is recommended that DR and 24HDR tools be repeated over several days. These recommendations might be difficult to apply, expensive and time consuming particularly in the setting of lack of literacy and old age, especially in large scale population-based studies [7–9].

FFQ is easier and less costly and can be administered to large cohorts. It usually can categorize and rank individuals by their usual frequency of food consumption. FFQ allows for comparison of food and nutrient intake across populations and countries with similar cultures and food availability [7, 8, 10–13] and can also describe frequency over a longer period of time, from a month to a year [10, 12–15]. When including portion size in addition to frequency, the FFQ allows the estimation of quantities consumed per day, thus allowing estimation of nutrient intakes, and dietary patterns [7, 12].

Each FFQ is constructed based on specific study objectives and target populations and needs to be validated to serve other groups and other study designs. For more reliable results, adaptation should therefore be made to the age-group targeted [12, 16]. The FFQ relies on the participant’s memory and conceptual skills that tend to decline with age [5, 8, 13, 17]. Errors related to cognitive functions and literacy should be accounted for when designing the validation study. Therefore, for more precision, it is preferred that the information be obtained from caregivers or proxy sources, when cognitive functions of an individual is diminished [8, 13, 14]. As opposed to self-administered questionnaire, the interview-based FFQ questionnaire does not rely on the literacy and numerical skills of the respondent, and is considered more suitable for dietary assessment with older adults [7].

Non-institutionalized older adults in Lebanon are susceptible to a higher risk of malnutrition and insufficient dietary intakes due to inappropriate or inexistant retirement plans, limited access to health care services and social welfare programs, as well as inadequate living conditions [2, 18]. This food insecurity has been aggravated by the Covid-19 pandemic, and the unprecedent political, social and economic crisis in Lebanon. It is therefore important to develop a tool for dietary assessment adapted to this sub-group of the population to better assess their nutritional intake and needs, and contribute in improving their health status and wellness.

Questionnaires used to evaluate food intake of older adults is scarce, particularly in the East Mediterranean region. In neighboring countries and in Lebanon, other questionnaires were used to estimate food and nutrient intake in adult population, but so far, no FFQ was validated exclusively among Lebanese older adults [11, 19–24].

The aim of our study is therefore to validate a quantitative FFQ, specifically tailored to evaluate nutrient consumption of community dwelling Lebanese, 60 years of age and older, using the 24HDR as a reference method. This questionnaire could serve for possible use in Lebanon and other Arabic speaking Mediterranean countries as well.

## Subjects and methods

### Study design and data collection

To study the validity of the FFQ, the mean of two 24HDR was used as a reference method, and to test the reproducibility of the FFQ, the same questionnaire was administered twice within a one-month period.

**Fig. 1:**
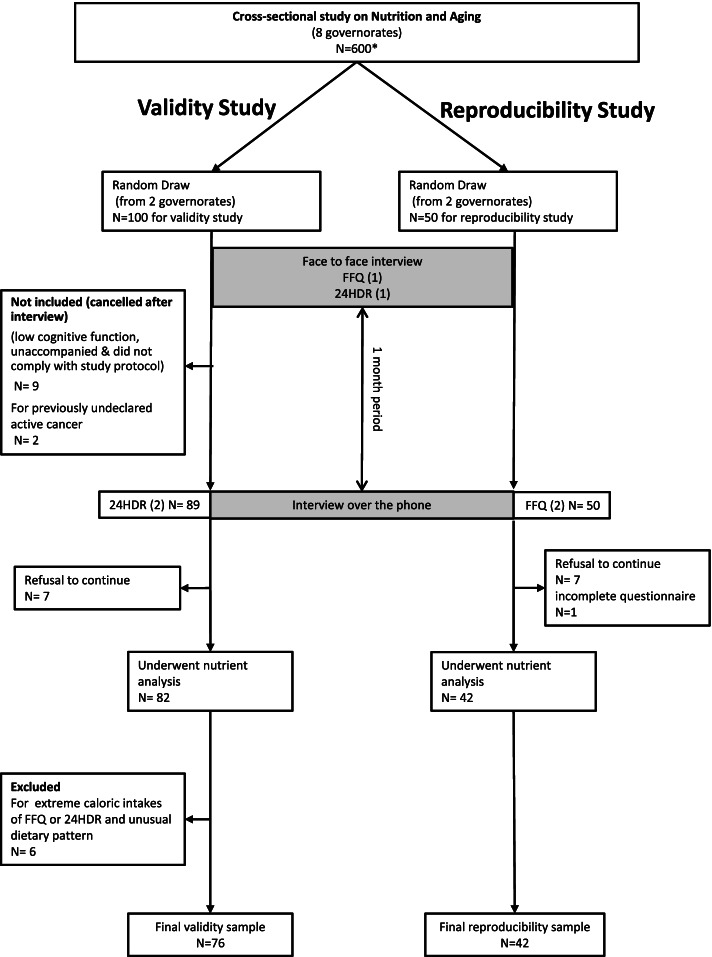
Design of the reproducibility and validity study of FFQ among older Lebanese. 24HDR: 24-hour dietary recall, FFQ: food frequency questionnaire

From January 2017 till June 2017, recruitment of participants and data collection were carried out in collaboration with the Ministry of Social Affairs (MOSA) through the medico-social centers that serve low to middle class income families. As illustrated in Fig. 1, the first meeting with the participant was thru a face-to-face interview. It lasted around 30-45 minutes and was performed in 2 phases: the first one included collection of general information (including cognitive tests), and dietary interview (FFQ and 24HDR), and the second included anthropometric measurements and other data in relation to health. A 10-minute break separated the 2 phases, to allow the participant to rest. The second interview with the participant, was performed within a one-month period, over the phone, and included both second FFQ and 24HDR. In our study, caretakers were interviewed when subjects were identified to have decline in cognitive functions assessed through the Mini-Mental State Examination (MMSE) [25] and “Test des Neufs Images” (TNI) [26]. In case of significant cognitive decline i.e., when participants scored at or below the population specific cut-off for MMSE or TNI total recall score lower or equal to 9, then the accompanying person, usually a relative living with the participant in the same household, was asked to fill the questionnaire on behalf of the participant. All interviewers were dietitians who received an extensive training before the start of data collection. Each person was interviewed at the MOSA center near his/her home, and for participants unable to attend, the interview was performed by the same research team at home.

To avoid inter-rater and reporting biases, participants were administered twice the FFQ and 24HDR by the same interviewer, and all filled questionnaires were reviewed by the principal investigator and field interviewers before data entry.

### Study sample

As illustrated by Fig. 1, the study samples were selected from our cross-sectional study on Nutrition and Aging. We included a randomly selected group of 50 and 100 participants respectively, aged 60 years and older, belonging to 2 of the 8 governorates of Lebanon. Individuals who were totally dependent, with known active cancer disease, undergoing dialysis, with advanced liver disease, with major hearing and visual problems, or receiving artificial nutritional support were not included in the study [27].

For both analyses, the size of samples was set for an expected intra-class correlation (ICC) value of 0.75 and a minimum acceptable value of 0.5, with 95% confidence and a desired power of 80%. The number of participants for the reproducibility study was 42 out of 50 initially selected for this analysis (*n =* 7 declining to do the second interview, and *n =* 1 for incomplete FFQ). A total of 13 out of 42 had their questionnaire answered by accompanying person, because of low cognitive test results. From an initial sample of 100 individuals included to test for the validity of the FFQ, and after a preliminary analysis, 24 participants were not included in the study: subjects with previously undeclared active cancer (*n =* 2), unaccompanied individuals with altered mental status and who did not comply with the study protocol (*n =* 9), those refusing to undergo the second interview (*n =* 7), those having extreme daily dietary intake (either < 600 Kcal/day (*n =* 1) or > 4000 Kcal/day (*n =* 2)), as well as those having extreme restrictions and may not be representing a regular feeding pattern (interview was performed during fasting for more than 14 hours period) (*n =* 3). A total of 34 out of 76 had their questionnaires answered by accompanying person, because of low cognitive test results. Retention rates within a one-month interval for repeated questionnaires, for FFQ and 24HDR, were respectively 84 and 76%.

### Development of the FFQ

Our initial questionnaire, including 113 food items, was developed based on foods commonly consumed by older Lebanese. Particular attention was made to include foods reflecting nutrients with potential link to cognitive decline and frailty [3, 28–35]. This questionnaire was tested on a small group of 10 individuals, followed by a discussion with research dietitians to adjust for misrepresentations of food items and for the final adjustment of the questionnaire. Redundant items and not frequently consumed items were excluded from the list. The final FFQ used for the validity and reproducibility studies included the usual consumption of 90 food items representing all food groups. As illustrated in Table 1, food categories included in the questionnaire are bread and cereals, milk and dairy products, vegetables and fruits, meat, poultry and fish, fats and oils, sweets and desserts, and non-alcoholic beverages, as well as some traditional local foods and dishes. Alcohol consumption was removed from the list because of a very low consumption rate in the studied sample. Foods were grouped based on similarity in their nutritional profiles and frequency of consumption. Consumption of these items were reported as usual portion size used daily, weekly, or monthly. A manual illustrating the usual portions of foods listed in the FFQ, with their respective weights, was developed for the study to help investigators and participants better estimate quantities ingested. Participants were asked to express their answers by describing their habitual food intake the previous year. The final portions consumed were then translated into daily consumption. To account for seasonal variability and based on availability of fruits and vegetables on the Lebanese market, a seasonal coefficient (S) was added to the list of fruits and vegetables. This coefficient is accounted for in the nutrient analysis by dividing consumption with the corresponding coefficient.

**Table 1:** Food groups and food items of the food frequency questionnaire

Food group	Food items included	Portion size used
Refined flour products	All types of bread including Lebanese bread, crisp bread, croissant, “Mankouche” (Lebanese dough-based product)	1 oz. of bread or ½ cup cooked cereals
Whole cereals and products	Whole-wheat, whole-wheat bread, oat breads and bulgur	
Rice	White rice	
Pizza and pasta	Bread equivalent of pizza, pasta	
Corn and breakfast cereals	Breakfast cereals and corn	
Potato	Potatoes and potato products	½ cup vegetable
Raw vegetables	Raw green leafy vegetables, tomatoes, cucumbers, mushrooms, sprouts and mixed salad/vegetables	
Cooked vegetables	Green beans, cooked cruciferous, zucchini, eggplants, carrots, pumpkins, bell peppers, sweet potatoes, green peas, onions, garlic	
Fruits	Fresh and dried fruits, fresh and bottled fruit juice	Usual portion size
Legumes	Lentils, chickpeas and beans, except soya	1 oz. of meat or equivalent
Meat & poultry	Beef, veal, lamb/mutton, goat, poultry, organ meat, processed meat from red meat or poultry (e.g. Ham, sausages)	
Eggs	Eggs boiled or fried	
Fish and shellfish	Fresh and canned fish, fish products and seafood.	
Vegetable oils	Vegetable oils including corn, peanut, sunflower, and canola oils, and mayonnaise	1 teaspoon equivalent of fat
Olive, seeds and oleaginous fruits	Olives, olive oil, sesame paste (“Tahini”) avocado, nuts	
Processed and saturated fats	Margarine, ghee and butter	
Milk & dairy products	Whole, light, or skimmed liquid milk, processed milk (condensed, dried), dairy dishes, cheeses	1 cup of milk equivalent
Low fat sweets	Wafers, “Meghle”, gelatin-based dessert, milk-based puddings	Usual serving size
High fat sweets	Oriental sweets and baklava, cakes and desserts, chocolate	
Sugars and jams	Sugar and confectionery sugar, jam, marmalade, honey,” Halawa”, soft drinks.	1 teaspoon of sugar

### 24HDR interview

Participants were asked to recall and describe in detail and in an open-ended manner the foods and beverages they consumed the previous day, starting from breakfast onwards. The interviewer first asked the participant if the day described was a usual day regarding his/her food consumption and dietary habits. The portion size consumed by the individual was estimated, as with the FFQ, using the portion manual guide, and standard measuring cups and spoons, to help estimate more accurately participant’s consumptions. For composite dishes, participants were always asked to describe the cooking method, the type and quantity of fat used for the whole household. For better analysis, as with the FFQ, brand names for specific foods such as milk, bread, biscuits, chocolates, and processed foods were also reported by the interviewer. As for beverages such as milk (from powder), coffee, tea and other drinks, methods of preparation were also detailed, and specific ingredients were added to the report.

### Nutrient intake analysis

Daily food consumption, reported by the FFQ was analyzed by the Nutrilog software (Nutrilog, version 3.2, France) to extract daily nutrient intake. The United States Department of Agriculture/ Standard reference 28 (USDA/SR28) and the Canadian Nutrient File (CNF 2015) databases were used to analyze nutrient composition of simple foods, and branded foods. For composite traditional dishes and some locally consumed desserts, not found in the database, recipes from known local cookbooks and pastry chefs were entered to the database and used in the nutrient analysis. Data on food consumed were reported simultaneously in (g) and as portions. The nutrient intakes of the two 24HDR were also extracted using the same methods.

### Nutrient adequacy

To analyze the concordance between FFQ and mean 24HDR in classifying individuals in terms of nutrient adequacy, we calculated The Nutrient Adequacy ratio (NAR) of 17 selected nutrients, including vitamins A, D, E, K, C, B1, B2, B3, B6, B9, B12, and minerals such as calcium, phosphorus, magnesium, iron, selenium and zinc. The NAR was calculated by dividing the estimated nutrient intake of individuals by the age and sex-specific recommended dietary allowance (RDA) for these nutrients, according to the established dietary reference intake (DRI) recommendations [36] For all nutrients, RDA values were used except for vitamin K, where adequate intake (AI) was used as the DRI value for comparison.

### Statistical analyses

IBM-SPSS 20.0 was used for statistical analysis. All macronutrients, 10 vitamins and 5 minerals estimated intakes were reported and compared for the validity and the reproducibility studies respectively. For numerical variables, data was expressed as mean ± standard deviation and median (interquartile ranges), and as numbers and percentages, for categorical variables. Student t-test and chi-square test were used to compare numeric data and categorical data respectively. Normal distribution of calories and nutrients was assessed using Kolmogorov-Smirnov tests, and data was processed accordingly. Characteristics of the reproducibility and the validation study samples were compared using Mann-Whitney U test, for continuous data and Chi-square test for categorical data. Wilcoxon signed-ranks tests were used for comparisons of both related samples of food frequency questionnaires, and for the difference between FFQ and mean 24HDR. For normally distributed data, Pearson’s correlation coefficient was used to test the reproducibility (test-retest) of the FFQ and the relation between estimated macro and micronutrients between the FFQ and 24HRs. For data that was not normally distributed, Spearman’s correlation coefficient was used. Correlation coefficients and their respective 95% confidence intervals (CI) were calculated and *p <* 0.05 was considered significant. To assess the internal consistency of the FFQ, after standardization of the variables, all items were computed in Cronbach’s alpha statistic. Alpha values between 0.7 and 0.8 indicate a good reliability, and a value higher than 0.9 would imply that all items on the questionnaire were very strongly related to each other and reliable for assessing the construct. To measure within-person variability, intra-class correlations (ICC) were calculated between the first and the second FFQ administration, using one-way random effects model, where people’s effects are random whereas interviewers performed under the same conditions. Values of r < 0.5 indicate a poor reliability, between 0.5 and 0.75 a moderate reliability, between 0.75 - 0.9 a good reliability, and greater than 0.9 excellent reliability [37]. Energy adjusted correlations between nutrients, estimated by FFQ and 24HDR, were calculated by pairwise linear regression models. Percentage agreement was calculated by the ratio of classification in the same or adjacent quartile to distant quartiles between 24HDR and FFQ, and through Kappa statistics. Bland-Altman plots were used to compare and visualize the agreement between 24HRs and FFQ, and both FFQ administrations.

To measure agreement of NAR estimation between the FFQ and the mean 24HDR methods, we calculated the percentage agreement between NAR estimated by FFQ and mean 24HDR by comparing classification in the same or adjacent quartile to distant quartiles, and through Kappa statistics. Concordance between the 2 NAR classifications was also measured using Spearman’s correlation coefficient.

## Results

### Characteristics of the samples

Characteristics of the sample population are presented in Table 2. Individuals who participated in the validity study (*N =* 76), had a mean age of 74.4 ± 6.9 years and a mean BMI of 30.3 ± 6.2 kg/m^2^. In this sample, 63.2% were women, 60.5% being married, and 84.2% living with their partners or relatives. The group of individuals included in the reproducibility/reliability study (*N =* 42) resembles the validity study sample, and characteristics of both did not differ statistically.

**Table 2:** Socio-demographic and anthropometric characteristics of the sample population for the validity and reproducibility study

Baseline characteristics		Validation sample *N =* 76	Reproducibility sample *N =* 42	*P* value
Anthropometric measurements	Age (y)	74 (74.4 ± 6.9)	72 (72.2 ± 5.8)	0.09
	BMI (kg/m^2^)	29.4 (30.3 ± 6.2)	26.9 (28.7 ± 6.1)	0.136
	WC (cm)	103 (104.6 ± 12.8)	103.8 (103.6 ± 13.5)	0.722
Gender	Male	28 (36.8%)	18 (42.9%)	0.521
	Female	48 (63.2%)	24 (57.1%)	
Living Conditions	Alone	12 (15.8%)	4 (9.5%)	0.629
	With partner	48 (63.2%)	29 (69%)	
	With family members	16 (21%)	9 (21.4%)	
Income	Insufficient	47 (61.8%)	2 (4.8%)	0.540
	Sufficient	29 (38.2%)	40 (95.2%)	
Marital Status	Married	46 (60.5%)	34 (81%)	0.158
	Divorced	4 (5.3%)	1 (2.4%)	
	Single	3 (3.9%)	1 (2.4%)	
	Widow/er	23 (30.3%)	6 (14.3%)	
Level of Education	Less than 7 years	36 (47.4%)	17 (40.5%)	0.471
	7 years and more	40 (52.6%)	25 (59.5%)	

*BMI* Body mass index, *WC* Waist circumference

Numeric data are represented as median and (mean ± SD), categorical data are shown as count and (percentages)

Student t-test and chi-square test were used to compare numeric data and categorical data respectively

Statistical significance was considered for *p <* 0.05

### Reproducibility study

The reproducibility study included 42 individuals, by repeating the FFQ questionnaire within a one-month interval. Median and mean intakes ± standard deviation (SD) of calories, macronutrients, and micronutrients of both FFQ1 and FFQ2 are reported in Table 3. Test and re-test means showed no significant differences between all paired macro- and micro-nutrient samples. The mean relative difference between both FFQ questionnaires was 1.03% ± 6.59%. Highest relative difference was noted for fiber intake (20.59%) and to a lesser extent for vitamin A intake (15.71%).

**Table 3:** Reproducibility study of food frequency questionnaire: mean ± (SD), median (IQR), intraclass correlation coefficients and correlation coefficients for energy and nutrients. (*N =* 42)

	FFQ1 Mean ± SD	FFQ2 Mean ± SD	FFQ1 Median (IQR)	FFQ2 Median (IQR)	***P*** value	Relative difference	ICC (95%-CI)	Correlation (95%-CI)
**Calories (Cal/d)**	2077 ± 635	2072 ± 645	1879.5 (1655-2668)	1851 (1641.5-2535)	0.88	0.24%	0.926 (0.863-0.960)	0.877 (0.782-0.932)
**Protein (g/d)**	72.9 ± 23.9	75.6 ± 23.3	68.2 (53.8-86.5)	70.5 (56.2-92.8)	0.30	−3.60%	0.906 (0.827-0.950)	0.848 (0.733-0.915)
**Fat (g/d)**	105.9 ± 35.2	104 ± 34.2	95.7 (77.48-125)	95.5 (81-119.5)	0.32	1.80%	0.908 (0.829-0.950)	0.768 (0.906-0869)
**CHO (g/d)**	215.2 ± 71.4	217.7 ± 72.4	204.5 (172-254)	199 (167.5-254)	0.77	−1.15%	0.862 (0.745-0.926)	0.784 (0.631-0.878)
**Sugar (g/d)**	62.6 ± 33.4	63.1 ± 30.4	51.7 (42.2-73.2)	53.6 (44-72.5)	0.35	−0.80%	0.888 (0.793-0.940)	0.720 (0.533-0.84)
**Fibers (g/d)**	20 ± 7.4	24.7 ± 30.2	17.9 (14.7-25.9)	20.2 (15.3-24.5)	0.37	−20.59%	0.366 (−0.174-0.658)	0.907 (0.833-0.949)
**SAFA (% TEI)**	12.8 ± 2.5	13.4 ± 2.5	12.4 (11-14.4)	13.4 (11.5-15.4)	0.07	−4.67%	0.763 (0.561-0.872)	0.634 (0.381-0.867)
**MUFA (% TEI)**	20.9 ± 3.7	20.2 ± 3.5	20.7 (18.2-23.3)	20.4 (17.3-22.3)	0.21	3.86%	0.523 (0.117-0.743)	0.361 (0.068-0.711)
**PUFA (% TEI)**	9.1 ± 1.7	8.4 ± 2	9 (7.7-10.23)	8.3 (6.9-9.9)	0.06	7.33%	0.496 (0.066-0.728)	0.362 (0.056-0.581)
**ω3 FA (g/d)**	1.1 ± 1	1 ± 0.8	0.8 (0.53-1.22)	0.7 (0.5-1.2)	0.19	13.73%	0.811 (0.649-0.898)	0.455 (0.176-0.666)
**ω6/ω3 FA ratio**	17.6 ± 6.7	17.3 ± 6.6	18.8 (13.1-22.3)	17.4 (12.7-21.3)	0.77	1.90%	0.645 (0.343-0.809)	0.468 (0.187-0.758)
**Cholesterol (mg/d)**	287 ± 152	274.1 ± 125	250.5 (186-351.5)	239 (189-326)	0.72	4.54%	0.603 (0.264-0.786)	0.383 (0.09-0.615)
**VIT A (μg/d)**	1080 ± 1389	922 ± 491	821.5 (497.5-1064)	865.5 (531-1096)	0.87	15.71%	0.402 (−0.107-0.678)	0.755 (0.586-0.861)
**VIT D (μg/d)**	2.3 ± 2.4	2.3 ± 2.2	1.3 (0.8-2.6)	1.3 (0.9-2.2)	0.45	0.30%	0.825 (0.675-0.905)	0.625 (0.397-0.78)
**VIT E (mg/d)**	12.7 ± 4.7	12 ± 4	13 (9-15.9)	11.3 (8.7-14.9)	0.13	5.55%	0.776 (0.586-0.880)	0.654 (0.437-0.799)
**VIT K (μg/d)**	314.6 ± 221.6	291 ± 159	259.5 (172.3-472.8)	265.5 (182.5-403.3)	0.44	7.91%	0.832 (0.689-0.910)	0.854 (0.73-0.919)
**VIT C (mg/d)**	89.6 ± 52.7	83.7 ± 46.7	75.9 (47.3-138.5)	71 (54.3-98.1)	0.38	6.84%	0.773 (0.581-0.878)	0.623 (0.394-0.779)
**VIT B1 (mg/d)**	1.1 ± 0.4	1.1 ± 0.4	1 (0.8-1.4)	1 (0.8-1.3)	0.99	−0.20%	0.857 (0.735-0.923)	0.804 (0.662-0.89)
**VIT B2 (mg/d)**	1.8 ± 0.7	1.8 ± 0.5	1.7 (1.3-2.1)	1.7 (1.5-2)	0.60	0.48%	0.725 (0.492-0.852)	0.634 (0.409-0.786)
**VIT B6 (mg/d)**	1.5 ± 0.6	1.5 ± 0.46	1.4 (1-1.8)	1.4 (1.2-1.8)	0.20	−3.29%	0.908 (0.830-0.951)	0.836 (0.714-0.908)
**VIT B9 (μg/d)**	366.4 ± 150.7	362.7 ± 146.2	328 (243.3-484.8)	329.5 (260.3-462)	0.92	1.01%	0.889 (0.795-0.940)	0.829 (0.702-0.904)
**VIT B12 (μg/d)**	6 ± 10	5.4 ± 3.4	4 (3-5.6)	4 (3.2-6.3)	0.43	10.53%	0.42 (−0.074-0.687)	0.621 (0.391-0.778)
**Magnesium (mg/d)**	357.3 ± 131.2	358.4 ± 110.1	350.5 (246-447.5)	354 (292.5-397.5)	0.82	−0.31%	0.773 (0.580-0.878)	0.736 (0.557-0.849)
**Calcium (mg/d)**	858.4 ± 289.6	909.6 ± 281.8	883.5 (593-1060)	844 (753-1047.3)	0.19	−5.79%	0.833 (0.692-0.910)	0.749 (0.577-0.857)
**Iron (mg/d)**	12.8 ± 4.9	12.69 ± 5	11.9 (8.9-16.8)	12 (9.6-14.4)	0.85	1.15%	0.913 (0.840-0.953)	0.843 (0.725-0.913)
**Zinc (mg/d)**	10.6 ± 3.7	11.1 ± 3.6	10.1 (8.2-12.8)	10.4 (8.7-13.6)	0.29	−4.74%	0.850 (0.722-0.919)	0.808 (0.669-0.92)
**Selenium (μg/d)**	91.5 ± 38.6	94.7 ± 33.5	85.7 (66-114)	94.6 (66.2-111)	0.74	−3.51%	0.811 (0.650-0.898)	0.707 (0.514-0.832)

*FFQ1* First food frequency questionnaire administration, *FFQ2* Second food frequency questionnaire administration, *ICC* Intraclass correlation, *Cal/d* Calories per day, *CHO* Carbohydrates, *SAFA* Saturated fatty acids, *MUFA* Mono-unsaturated fatty acids, *PUFA* Poly-unsaturated fatty acids, *% TEI* Percentage from total energy intake, *ω3 FA* Omega-3 fatty acids, *ω6/ω3 FA ratio* Omega 3 to omega 6 fatty acids ratio, *Vit* Vitamin, *IQR* Interquartile range, *SD* Standard deviation, *CI* Confidence interval

Intake values of both FFQ administrations are represented as mean ± standard deviation and median (interquartile range)

Relative difference = mean (FFQ1 - FFQ2)/ mean (FFQ1&FFQ2) *100

For normally distributed variables, parametric tests including paired t test and Pearson correlation were performed. Nonparametric tests, Wilcoxon and Spearman’s correlation were performed otherwise

Statistical significance was considered for *p <* 0.05

To measure within- person variability, ICC and correlation coefficients (Spearman’s or Pearson’s, depending on data normality) were calculated comparing the two administrations of the FFQ. Based on ICC, a good and excellent reliability was noted for caloric intake and most macronutrients. Remaining nutrients had a moderate to good reliability except for PUFA, vitamins A, B12 and fibers. Correlation coefficients for fibers, vitamin A and B12 showed a strong association between both administrations, as found for caloric intake, all macronutrients and micronutrients. Fair correlation (*r =* 0.3-0.5) was noted for MUFA, PUFA, omega-3 fatty acids (ω3 FA), omega 6 to omega 3 fatty acids ratio (ω6/ω3 ratio) and cholesterol intakes.

Internal consistency, measured by Cronbach Alpha based on standardized items, was respectively 0.947 and 0.962 for the first and second administrations of the questionnaire, and their mean ICC, was respectively *r =* 0.82 (95% CI = 0.792-0.846) and *r =* 0.845 (95% CI = 0.77-0.905).

### Validity study

Caloric intake and intake of macronutrients and some relevant micronutrients estimated by the FFQ, were compared to the mean of both 24HDRs, allowing to evaluate the accuracy of the FFQ in our study sample and estimate its agreement with this chosen standard. Details of mean and median daily intake of nutrients and their correlations are shown in Table 4. For most nutrients, mean values obtained by the FFQ tended to be most of the time, significantly higher than values obtained by the mean 24HDR. The mean relative difference between the 2 methods was (19.5% ± 20.2%) showing an estimation ranging in absolute values between 3.1% for MUFA and 84% for vitamin B12, with differences confirmed by Mann-Whitney U test, for most nutrients.

**Table 4:** Criteria validity: degree of association and level of agreement between average daily nutrient intakes estimated by the FFQ and the mean of 24HDR. (*N =* 76)

	FFQ Mean ± SD Median (IQR)	24HDR Mean ± SD Median (IQR)	***P*** value^**a**^	% Difference	% Agreement	Kappa coefficient	Correlation (Energy adjusted)
**Calories (Cal)**	1739 ± 5,331,686 (1387-2030)	1433 ± 3,871,371 (1126-1708)	< 0.001	19.3	88.2	0.75	
**Protein (g)**	58.4 ± 20.8 56.2 (41. 6-69.2)	47.2 ± 15.3 43.5 (35.7-58.8)	< 0.001	21.1	81.6	0.62	0.626^b^
**Fat (g)**	84.6 ± 27.4 79.8 (68.2-102)	66.7 ± 21.2 63.3 (49.9-81.2)	< 0.001	23.6	86.8	0.73	0.595^b^
**CHO (g)**	196 ± 71.2188 (152-219)	167 ± 54.6154 (128-201)	< 0.001	16.4	85.5	0.70	0.719^b^
**Sugars (g)**	66.5 ± 32.7 60.4 (46.5-79.4)	44.4 ± 23.13 38.5 (28.3-53.6)	< 0.001	39.9	90.8	0.82	0.66^b^
**Fibers (g)**	19.3 ± 7.5 18.7 (14-22.6)	17.5 ± 8 16.1 (12.5-20)	0.071	9.5	69.7	0.39	0.56^b^
**SAFA (% TEI)**	12.4 ± 3 12.1 (10.7-14.7)	11 ± 3.7 10.9 (9.1-12.5)	< 0.001	11.7	73.7	0.47	0.36^b^
**MUFA (% TEI)**	20.2 ± 4.1 19.9 (17.5-23.4)	19.6 ± 5.5 19.6 (15.6-23.6)	0.199	3.1	80.3	0.60	0.446^b^
**PUFA (% TEI)**	8.4 ± 2.6 8 (6.8-9.6)	9.3 ± 4.1 8.9 (6.3-10.6)	0.081	−10.3	71.1	0.41	0.347^b^
**ω3 FA (g)**	0.85 ± 0.61 0.69 (0.41-1.11)	0.74 ± 0.69 0.53 (0.29-0.81)	0.010	13.7	80.3	0.61	0.519^b^
**ω6/ω3 FA ratio**	15.1 ± 8.5 12.6 (8.7-21.3)	18.3 ± 10.6 15.4 (9.5-24.6)	0.016	− 19.3	76.3	0.51	0.519^b^
**Cholesterol (mg)**	218 ± 123.4197 (120.5-294.3)	128.2 ± 105.6 85.4 (60.9-159)	< 0.001	51.9	73.7	0.48	0.43^b^
**VIT A (μg)**	905.1 ± 790.6677 (435-1135)	431.4 ± 320.1361 (203-539)	< 0.001	70.9	69.7	0.40	0.269
**VIT D (μg)**	1.4 ± 1.1 1.1 (0.7-1.7)	0.9 ± 1.2 0.5 (0.2-1.1)	< 0.001	9	76.3	0.53	0.459^b^
**VIT E (mg)**	11.3 ± 4.3 11.1 (8.6-13.8)	10 ± 4.6 9.2 (6.36-12)	0.001	13	82.9	0.65	0.602^b^
**VIT K (μg)**	271 ± 188,233 (127-365)	262 ± 317 88.3 (41.8-5)	0.206	3.2	73.7	0.48	0.413^b^
**VIT C (mg)**	98.2 ± 52.4 85.8 (62.7-1)	90.4 ± 63.4 75.6 (44.7-1)	0.286	8.3	81.6	0.63	0.443
**VIT B1 (mg)**	0.9 ± 0.3 0.93 (0.7-1.1)	0.9 ± 0.3 0.78 (0.6-1.1)	0.009	8.8	81.6	0.62	0.634^b^
**VIT B2 (mg)**	1.5 ± 0.6 1.5 (1.1-1.8)	1.1 ± 0.4 1 (0.8-1.2)	< 0.001	36.2	76.3	0.53	0.529^b^
**VIT B3 (mg)**	17.2 ± 6.6 17.2 (12.8-21.1)	15.1 ± 6.3 14.5 (11.8-17.4)	< 0.001	13.1	86.8	0.73	0.544^b^
**VIT B6 (mg)**	1.4 ± 0.47 1.4 (1-1.7)	1.12 ± 0.45 1.1 (0.8-1.3)	< 0.001	22.3	78.9	0.58	0.669^b^
**VIT B9 (μg)**	316 ± 129,296 (217-438)	285 ± 157,264 (173-360)	0.023	10.2	71.1	0.41	0.522^b^
**VIT B12 (μg)**	5.3 ± 6. 3.6 (2.5-5.6)	2.2 ± 2.1 1.8 (0.9-3)	< 0.001	84	61.8	0.24	0.17
**Magnesium (mg)**	324 ± 113,315 (244-393)	286 ± 109,265 (211-350)	0.002	12.5	80.3	0.60	0.638^b^
**Calcium (mg)**	695 ± 289,701 (475-914)	489 ± 217,468 (303-637)	< 0.001	34.8	84.2	0.68	0.568^b^
**Iron (mg)**	10.5 ± 3.8 10 (7.5-12.5)	9.7 ± 4.1 9.5 (6.3-11.8)	0.101	7.9	73.7	0.47	0.571^b^
**Zinc (mg)**	8.9 ± 3.3 8.3 (6.7-10.5)	7.04 ± 2.7 6.4 (5.2-8.5)	< 0.001	23.5	75	0.48	0.639^b^
**Selenium (μg)**	78.2 ± 27.6 74.7 (59.9-97.5)	64.3 ± 35.2 56.8 (41.3-86.6)	< 0.001	19.5	80.3	0.61	0.594^b^

*Abbreviations: FFQ* Food frequency questionnaire, *24HDR* Twenty-four hours dietary recall, *%* Percentage, *Cal/d* Calories per day, *CHO* Carbohydrates, *SAFA* Saturated fatty acids, *MUFA* Mono-unsaturated fatty acids, *PUFA* Poly-unsaturated fatty acids, *% TEI* Percentage from total energy intake, *Vit* Vitamin, *ω3 FA* Omega-3 fatty acids, *ω6/ω3 FA ratio* Omega 3 to omega 6 fatty acids ratio, *IQR* Interquartile range

Relative percentage difference was calculated as (FFQ1 – Mean 24HDR)/Mean 24HDR) X100

^a^Wilcoxon signed rank test, significant with *p <* 0.05

^b^Correlations significant with *p <* 0.01

^c^Correlations significant with *p <* 0.05

Interrater reliability between FFQ and 24HDR was evaluated thru percentage agreement and Kappa statistics for all listed nutrient intakes. Percentage agreement between the two tools, calculated by the ratio of classification in the same or adjacent quartile, to distant quartiles, placed older individuals in the same or adjacent quartiles, on average, in 80% of the sample population, with agreements ranging from 90.8% for sugars to 61.8% for vitamin B12. Based on Kappa coefficient, agreement between FFQ and 24HDR was, strong (> 0.61), for total calories, total fat, sugars, vitamins E, C, B1, B3 and calcium. Agreement was moderate (0.41-0.6) for all remaining nutrients except for vitamins A, B12 and fibers with poor agreement. Mean Kappa value was 0.56.

Energy adjusted correlation between FFQ and mean 24HDR was performed by pairwise linear regression models and shown in Table 4. After adjustment, correlation coefficients of almost all nutrients showed improvement. Correlation was weak (r < 0.3) and non-significant only for vitamins A and B12, fair (0.3 -0.5) for SAFA, MUFA, PUFA, vitamins K and C, and moderate (0.5-0.7) for the remaining nutrients except for carbohydrates with excellent correlation. Adjusting for intake of nutrient-dense foods improved the agreement for vitamins A and B12, to 0.49 and 0.56 respectively.

### Bland-Altman analyses

**Fig. 2:**
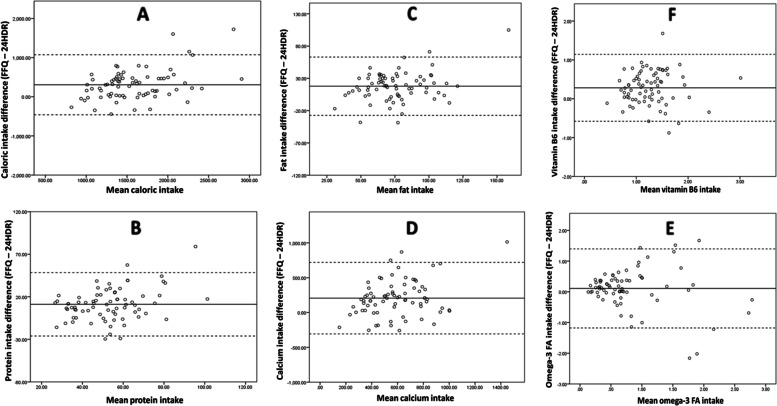
Agreement between average nutrient intakes measured by the FFQ and the mean of 24HDR. Agreement is measured by Bland–Altman plots for (**A**) caloric intake (Calories/day), (**B**) protein (g/day), (**C**) fat (g/day) and (**D**) calcium (mg/day), (**E**) omega 3 fatty acid (g/day), (**F**) vitamin B6 (mg/day). (—) lines represent mean difference (FFQ – mean 24HDR) and (− − -) represent lower and upper 95% limits of agreement. The higher and lower extremes represented the mean ± 2 SD

We selected a group of nutrients to evaluate the bias between the nutrient mean differences and determine the agreement interval of the differences, between the FFQ and reference method we used. This agreement, performed thru Bland-Altman analyses, is represented in Fig. 2, for calories, protein, fat, ω3 FA, selenium, and vitamin B6 intakes. Plots of other nutrients resembled those presented below (plots not shown). As shown in Fig. 2, the Y-axis represented the difference between FFQ and mean 24HDR, and the X-axis represented the mean of both tools for respective nutrients. The higher and lower extremes represented the mean ± 2 SD or the interval for the limits of agreement within which almost all our subjects fell within. The limits of agreement were adequate, and values were scattered evenly. (We also performed Bland-Altman plots, analyzing agreement between the first and second administrations of the FFQ (data in supplementary material)).

### Concordance of NAR between FFQ and mean 24HDR

Table 5 shows the degree of agreement between FFQ and mean 24HDR in classifying individuals for nutrient adequacy of the list of selected nutrients. As previously shown, the FFQ compared to 24HDR tends to overestimate nutrient intakes as well as nutrient adequacy. Percentage agreement between 24HDR and FFQ for classifying intake with regard to nutrient adequacy, showed that these tools placed older individuals in the same or adjacent quartiles, on average, in 78.2% of the sample population, with agreements ranging between 85.5% for vitamin B1 to 60.5% for vitamin B12. Based on Kappa value, agreement was fair for vitamin B12, moderate for vitamins A, D, K, C, B2, B9, zinc and iron, and good for vitamins E, B1, B3, B6, magnesium, calcium and phosphorus.

**Table 5:** Concordance and Agreement between NAR of selected nutrients estimated by FFQ and mean 24HDR. (*N =* 76)

Nutrients	NAR 24HDR Median [IQR]	NAR FFQ Median [IQR]	% Agreement between same or adjacent quartile	Kappa	NAR Spearman Correlation	***P*** value^**b**^
**VIT A**	41.4 [27.8 - 76.2]	91.4 [55.7 - 154.1]	72.4%	0.447	0.152	0.016
**VIT D**	2.3 [1 - 6.3]	6.3 [3.7 - 9.5]	76.3%	0.526	0.457	< 0.01
**VIT E**	61 [42.8 - 79.2]	73.7 [57.3 - 92]	84.2%	0.684	0.623	< 0.01
**VIT K**^**a**^	87.9 [39.9 - 484]	218.3 [141.7 - 386.1]	76.3%	0.526	0.366	< 0.01
**VIT C**	93.6 [56.3 - 157.7]	111.2 [76.9 - 155.3]	77.6%	0.553	0.439	< 0.01
**VIT B1**	71.2 [52.7 - 93.9]	82.5 [63.6 - 99.1]	85.5%	0.71	0.553	< 0.01
**VIT B2**	86 [70.8 - 108]	130.8 [100.5 - 154.1]	77.6%	0.553	0.412	< 0.01
**VIT B3**	100 [74.5 - 118.3]	115.6 [85.6 - 144]	82.9%	0.658	0.557	< 0.01
**VIT B6**	67.4 [49.7 - 87]	85.7 [68.2 - 107.3]	84.2%	0.684	0.529	< 0.01
**VIT B9**	66 [43.3 - 89.4]	73.9 [54.5 - 107.6]	72.4%	0.447	0.349	< 0.01
**VIT B12**	73.5 [39.3 - 123.4]	150.4 [106 - 232.3]	60.5%	0.211	0.166	0.151
**Magnesium**	78.7 [55.2 - 102.8]	91.9 [69.2 - 111.7]	84.2%	0.684	0.593	< 0.01
**Calcium**	39.4 [25.3 - 53.3]	58.5 [39.8 - 76.5]	82.9%	0.658	0.506	< 0.01
**Phosphorus**	99.1 [80.5 - 130.3]	135.6 [97.9 - 156.7]	80.3%	0.605	0.429	< 0.01
**Iron**	118.8 [78.8 - 147.1]	125.1 [94.8 - 156.3]	76.3%	0.526	0.402	< 0.01
**Zinc**	71.8 [56.6 - 100]	89.5 [71.5 - 117.5]	76.3%	0.526	0.433	< 0.01
**Selenium**	103.3 [75.2 - 157.3]	135.8 [109.3 - 176.5]	78.9%	0.579	0.49	< 0.01

*Abbreviations: NAR* Nutrient adequacy ratio, *FFQ* Food frequency questionnaire, *24HDR* Twenty-four hours dietary recall, *%* Percentage, *Vit* Vitamin, *IQR* Interquartile range, *CHO* Carbohydrates

NAR was calculated as (nutrient estimated actual intake/ age and sex-specific nutrient RDA)

^a^ NAR for vitamin K was calculated using adequate intake (AI)

^b^ significant with *p <* 0.05

## Discussion

The present study showed that the proposed 90 food-items FFQ gives a good estimation of nutrient intake of older individuals in an Arabic-speaking Mediterranean country. The analysis showed that the FFQ has a good reproducibility and reasonable validity in relation to the mean of two 24HDR.

The results obtained in our repeatability study showed that all pairs of intakes estimated by the 2 FFQ administrations showed no significant difference for most macronutrients and micronutrients. FFQ administrations showed excellent internal consistency. The ICC analysis of the FFQ showed good reliability. For most nutrients, these findings align well with those reported in the meta-analysis on the reproducibility of FFQ by Cui et al., where the median of ICCs for the elderly (> 50 years) was good, with ICC coefficients ranging from 0.482 to 0.866, lower than those for younger age-groups for 22 of 40 nutrients analyzed. [38]. When comparing both FFQ administrations, our results showed good to strong correlation for almost all nutrients. Lower correlation coefficients, found for fatty acids and fiber intakes, could be explained by the fact that some participants might have been observing fasting (selective food exclusion, without reporting it), during which the animal products are excluded from their diet, and this might have affected their answers, rendering lower correlation coefficients for the aforementioned nutrients [39]. Moreover, social desirability bias related to healthy food consumption might have caused individuals to overreport such foods [40].

In our validity study, the percentage difference between the two tools showed a mean percentage difference between the FFQ and the reference method of 19.45%, with FFQ overreporting nutrient intake. As often reported, when comparing the FFQ to the mean of 24HDR, FFQ tends to overestimate nutrient intake for almost all nutrients [12, 19, 41, 42].

Agreement between FFQ and 24HDR was measured by first ranking participants’ dietary intake in quartiles, then assessing the percentage agreement between the same or adjacent quartiles and calculating Cohen’s Kappa coefficient and correlation coefficients. We were able to show that the used instruments are highly concordant. For most of nutrients, our FFQ ranked correctly around 80 and 78% of individuals, in the same or adjacent quartile as the mean 24HDR, whether in classifying nutrient intake or nutrient adequacy respectively. Moreover, Kappa coefficients detected moderate to good agreement between nutrient estimation and adequacy classification of participants between FFQ and mean 24HDR. Our results are concordant with previous reports where it was established that 50% of subjects should be correctly classified, and weighted kappa values should be above 0.4 for nutrients of interest [43, 44].

Our findings showed that FFQ overestimated intakes when compared to 24HDR, and correlation improved when adjustment for calories is made. Energy-adjusted nutrient intake estimated by FFQ compared to mean 24HDR, indicated correlation coefficients higher than 0.5 for all macronutrients and most micronutrients. A fair correlation only occurred with vitamins A, and B12. As reported by other studies, vitamin A intake is usually difficult to assess by FFQ, probably because of double counting of items, uneven distribution of vitamin A across food items, seasonal changes in dietary habits, social desirability bias, and overreporting of healthy nutrient-dense foods [8, 15, 40, 45, 46]. Adjustment with specific foods confirmed that the consumption of nutrient-dense foods as beef or sheep liver, reported in the 24HDRs, boosted the intake of these two nutrients and affected correlation. By adjusting for nutrient-dense food consumption, the correlation coefficients increased to a satisfactory level of approximately 0.5. Although extreme cases were excluded, as previously reported, a lower food diversification and a lower intake of foods from animal source during one of the 24HDRs, could further decrease vitamin A and B12 intakes thus affecting the correlation between the 2 dietary evaluation tools [39].

The main strength of our study was the consideration of age-related cognitive decline while assessing the validity and reproducibility of the FFQ, performed in a homogeneous older population. Previous reports in elderly population found errors in evaluating total energy intake related to cognitive ability, with an inverse association between reported calories and cognitive functions [47, 48]. In this age group, dietary recall can be biased with memory decline starting at age of 55 years. Consequently, age-related physical and mental impairments should be assessed to exclude individuals with possible deficit, to decrease reporting bias and improve validity [8]. In our study, we used close relatives to report consumption when cognitive decline was suspected. As reported previously, when assessing dietary intake, methodological adaption using caregivers instead of individuals themselves, was found to have moderate agreement for most estimated nutrients and food groups [14, 49].

To our knowledge, no previous validated questionnaire was developed to specifically describe and evaluate dietary habits of older individuals in the region.

This study has few limitations. To assess intake over 1 year, 4 days recall (1 day per season) are recommended to rule out seasonality bias [12]. Our choice was set at two 24HDR, because elderly population had a low response rate to repeated interviews and a high risk of attrition and drop-outs. Participation rates in surveys has been shown to be relatively low and to vary depending on the social setting, advancing age, altered health status, constraints in physical functions and impairment of cognitive functions and fatigue during long interviews, ranging between 37 to 79% response rates [50–53]. Furthermore, despite obtaining good agreement between FFQ and 24HDR, for most nutrients, the use of 24HDR as a reference is known to have intrinsic errors [7]. 24HDR requires a satisfactory memory and an adequate reporting. Some risk factors associated with underreporting include a lower educational level, the presence of obesity and female gender [8, 54–56]. In addition to that, the strict measure in selection criteria related to cognitive evaluation led to a lower number of valid questionnaires and decreased our sample sizes.

Special consideration should be given for seasonality that might affect older adults reporting on their food intake. We suggest repeating the FFQ bi-annually to represent more specifically habitual intake. A self-reported version of the FFQ questionnaire could be tested for validation to allow a wider use of the questionnaire among literate elderly participants in the future.

In this study we were able to test the validity and the reliability of a culture specific FFQ adapted to the elderly population in the Middle East Mediterranean region as compared to mean of two 24HDR and confirm that the FFQ remains one of the most suitable and inexpensive choices to describe food intake of elderly people of low economic status [57].

## Conclusion

Evaluation of food intake in elderly individuals is quiet challenging, and few countries in the Middle East developed and validated questionnaires in people aged 60 years and more. Our questionnaire showed satisfactory reliability and validity and could be suggested for a broader application as a suitable tool to estimate and evaluate dietary habits and nutrient intakes of older individuals living in Lebanon and in the East Mediterranean Arabic speaking countries.

## Supplementary Information

**Additional file 1.**

## Data Availability

All data generated or analyzed during this study are included in this published article [and its supplementary information files].

## Abbreviations

% TEI: Percentage from total energy intake
24HDR: 24-hours dietary recall
AI: Adequate intake
BMI: Body mass index
CHO: Carbohydrates
CI: Confidence interval
DR: Diet record
FFQ: Food frequency questionnaire
ICC: Intra-class correlation
IQR: Interquartile range
MMSE: Mini-mental state examination
MOSA: Ministry of social affairs
MUFA: Mono-unsaturated fatty acids
NAR: Nutrient adequacy ratio
PUFA: Poly-unsaturated fatty acids
RDA: Recommended dietary allowance
SD: Standard deviation
TNI: Test des neuf images
ω3 FA: Omega-3 fatty acids
ω6 FA: Omega-6 fatty acids
WC: Waist circumference
