# Supplementary material for: Validity and reliability of a food frequency questionnaire for community dwelling older adults in a Mediterranean country: Lebanon
Source: Nutr J. 2022 Jun 18;21:40. doi: 10.1186/s12937-022-00788-8 (PMC9206140; doi:10.1186/s12937-022-00788-8)
Supplement: Supplementary file 1 — Additional file 1. [file 12937_2022_788_MOESM1_ESM.pdf]

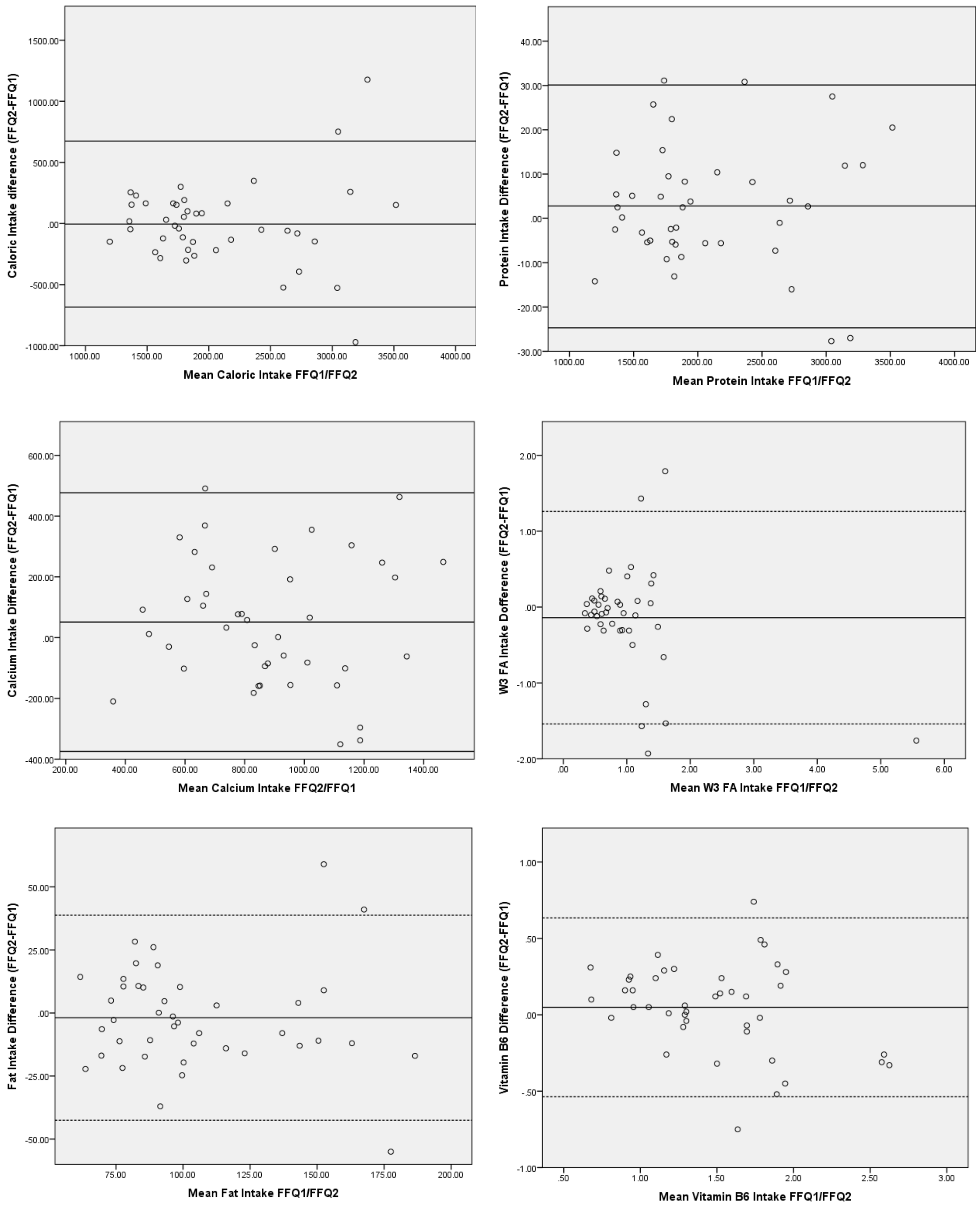

Figure S1. Bland–Altman plots showing agreement between estimated nutrient intakes (FFQ2 – FFQ1) against mean FFQ1/FFQ2 for (A) caloric intake (Calories/day), (B) protein (g/day), (C) fat (g/day) and (D) calcium (mg/day), (E) omega 3 fatty acid (g/day), (F) vitamin B6 (mg/day).
(—)Lines represent mean difference and (- - -) represent lower and upper 95% limits of agreement.
